# Pathomic model based on histopathological features and machine learning to predict IDO1 status and its association with breast cancer prognosis

**DOI:** 10.1007/s10549-024-07350-6

**Published:** 2024-05-23

**Authors:** Xiaohua Zhuo, Hailong Deng, Mingzhu Qiu, Xiaoming Qiu

**Affiliations:** Department of Pathology, Longyan First Affiliated Hospital of Fujian Medical University, Longyan, Fujian China

**Keywords:** Pathomic model, Histopathological feature, IDO1, Prognosis, Immunotherapy, Breast cancer

## Abstract

**Purpose:**

To establish a pathomic model using histopathological image features for predicting indoleamine 2,3-dioxygenase 1 (IDO1) status and its relationship with overall survival (OS) in breast cancer.

**Methods:**

A pathomic model was constructed using machine learning and histopathological images obtained from The Cancer Genome Atlas database to predict IDO1 expression. The model performance was evaluated based on the area under the curve, calibration curve, and decision curve analysis (DCA). Prediction scores (PSes) were generated from the model and applied to divide the patients into two groups. Survival outcomes, gene set enrichment, immune microenvironment, and tumor mutations were assessed between the two groups.

**Results:**

Survival analysis followed by multivariate correction revealed that high IDO1 is a protective factor for OS. Further, the model was calibrated, and it exhibited good discrimination. Additionally, the DCA showed that the proposed model provided a good clinical net benefit. The Kaplan–Meier analysis revealed a positive correlation between high PS and improved OS. Univariate and multivariate Cox regression analyses demonstrated that PS is an independent protective factor for OS. Moreover, differentially expressed genes were enriched in various essential biological processes, including extracellular matrix receptor interaction, angiogenesis, transforming growth factor β signaling, epithelial mesenchymal transition, cell junction, tryptophan metabolism, and heme metabolic processes. PS was positively correlated with M1 macrophages, CD8 + T cells, T follicular helper cells, and tumor mutational burden.

**Conclusion:**

These results indicate the potential ability of the proposed pathomic model to predict IDO1 status and the OS of breast cancer patients.

**Supplementary Information:**

The online version contains supplementary material available at 10.1007/s10549-024-07350-6.

## Introduction

In 2020, breast cancer emerged as the most prevalent type of cancer worldwide with approximately 2.3 million new cases and a staggering number of over 680,000 deaths [1]. The primary approaches for battling breast cancer typically involve surgical procedures and regimens such as radiation, chemotherapy, hormone treatments, and targeted therapies. Endocrine therapy is commonly employed for hormone receptor-positive tumors; however, resistance development is a common issue. For HER2-positive tumors, targeted therapy is often the preferred treatment approach. Few treatment options are available for hormone receptor-negative and HER2-negative breast cancers. Recently, immunotherapy has become a promising strategy against cancer by reactivating the immune system.

Blocking the immunosuppressive effects of indoleamine 2,3-dioxygenase 1 (IDO1) has emerged as a promising immunotherapeutic approach. IDO1 is an enzyme that converts tryptophan, an essential amino acid, into N-formyl-kynurenine. Although IDO1 is primarily expressed in mature dendritic cells, its expression is minimal or absent in regular tissues; however, it is inducible in most tissues. [2]. Numerous studies have demonstrated that IDO1 overexpression in tumors correlates with an unfavorable prognosis in several tumors, including esophageal squamous cell carcinoma [3], anal squamous cell carcinoma [4], and extrahepatic bile duct carcinoma [5]. Unexpectedly, Fang reported that higher IDO1 expression in breast cancer is associated with better overall survival (OS) [6]. The tumorigenic effects of IDO1 are mediated through multiple mechanisms, including the shaping of a tumor-favorable immune microenvironment due to tryptophan shortages and the accumulation of tryptophan catabolites [2, 7, 8], as well as activation of pro-tumorigenic signaling pathways such as PI3K/AKT signaling and the translocation of β-catenin from the cytoplasm into the nucleus [9, 10]. These findings imply that IDO1 can be a potential target for immunotherapy in cancers with IDO1 positivity. Therefore, extensive research has been conducted on IDO1 inhibitors through clinical trials to explore their potential for enhancing cancer immunotherapy [11, 12]. IDO1 inhibition could reverse the immunosuppressive effects of IDO1 and improve breast cancer outcomes [13–15]. Therefore, the sensitivity and accuracy of IDO1 detection are particularly important.

In current clinical practice, IDO1 status is determined through visual examinations of stained slides using immunohistochemical (IHC) assays. However, the IHC staining procedure is costly and time-intensive, and the test results could vary with differences in tissue preparation, antibodies, technician skill levels, and subjective interpretations of pathologists. Histopathologic image features derived from computer-aided pathological analyses have been used to make diagnostic assessments [16–19], prognostic predictions [20–22], and evaluate molecular expression levels [23–26] in breast cancer. Histological images were obtained from formalin-fixed tissue sections embedded in paraffin and stained with hematoxylin and eosin (H&E), which are widely used for pathologic diagnosis. H&E images could be easily obtained without the disadvantages of IHC staining.

In this study, we first identified the IDO1 expressions correlated with patient survival through bioinformatics analysis. Through our investigations on IDO1 in breast cancer, as well as its well-established immunomodulatory characteristics and the advantages of machine learning (ML) methods, we hypothesized that H&E image features could be useful for IDO1 status and outcome prediction in breast cancer; this approach remains unexplored. To test this hypothesis, we constructed an ML model, called the "pathomic model," using H&E image features extracted in The Cancer Genome Atlas (TCGA) database. Subsequently, the model performance was examined, and the potential mechanisms were explored. Our findings indicated that the pathomic model could be an easy‐to‐use surrogate for the assessment of IDO1 status, which might facilitate more objective, accurate, robust, and less expensive clinical decision-making.

## Materials and methods

### Data acquisition

First, data on breast cancer patients (*n* = 1,097) were downloaded from the TCGA database. The following patients were excluded: males (*n* = 12), those who are not newly diagnosed or treatment naïve (*n* = 15), those with missing survival data (*n* = 1), those with a survival duration of less than one month (*n* = 49), those with incomplete clinical data (*n* = 52), and those with lost expression data (*n* = 40). After applying the exclusion criteria, 928 patients were included here. H&E histopathological images (*n* = 1062) were obtained from the TCGA database. Low-quality images (*n* = 120) were eliminated, leaving 942 patients. Finally, the intersection of the two samples was considered, and 791 patients with RNA-seq data, complete clinical information, and qualified pathological images were included. Supplementary Fig. S1 shows the inclusion and exclusion criteria.

### Image segmentation and image feature extraction

To facilitate feature extraction, we employed Otsu's thresholding algorithm (accessible from https://opencv.org/) to segment whole slide images. Initially, images at 20 × magnification were divided into small sub-images with dimensions of 1024 × 1024 pixels, whereas images at 40 × magnification were divided into small sub-images with dimensions of 512 × 512 pixels and then upsampled to 1024 × 1024 pixels. Subsequently, pathologists reviewed each sub-image to remove images considered of poor quality (e.g., images with contamination, blurriness, or exceeding 50% white space). Thereafter, 10 sub-images per patient were randomly selected for further analysis.

Next, we used the PyRadiomics library in Python (https://pyradiomics.readthedocs.io/en/latest/) to extract features from each sub-image. In total, 93 original features (including first- and second-order features) and higher-order features (including Wavelet [LL, LH, HL, HH], LoG [kernel size: 1, 2, 3, 4, 5], Square, SquareRoot, Logarithm, Exponential, Gradient, and LBP2D) were extracted. Consequently, 1488 image features were derived per sub-image. To obtain deeper insights, further investigations were conducted by calculating the mean value of the 10 sub-images for each patient as their pathomic feature value.

### Screening of image features and model construction

First, the maximum-relevance minimum-redundancy (mRMR) algorithm was employed to eliminate redundant and irrelevant features. This algorithm ranked the input pathomic features by maximizing their predictive ability while minimizing the mutual information among features and was implemented using the mRMRe R package (https://cran.r-project.org/web/packages/mRMRe/index.html). Second, the recursive feature elimination (RFE) algorithm (https://www.rdocumentation.org/packages/caret/versions/6.0-92/topics/rfe) was applied to select important features and eliminate unimportant ones. This algorithm assessed the importance of each feature and ranked them according to their importance in model prediction and was implemented in the classification and regression training (caret) *R* package (https://cran.r-project.org/web/packages/caret/index.html). Third, the selected important features were used for ML prediction model building using a gradient boosting machine (GBM) (https://cran.r-project.org/web/packages/gbm/index.html) algorithm. This algorithm iteratively combined multiple weak decision tree learners through boosting to develop a robust predictive model. The GBM approach was implemented using the caret *R* package (https://cran.r-project.org/web/packages/caret/index.html).

### Performance evaluation

Data of 791 patients were randomly classified into training (*n* = 555, 70%) and validation (*n* = 236, 30%) sets. Each feature in the training set was standardized using a *z*-score. Each feature in the validation set was standardized using the average and standard deviation values obtained from the training set, and the differences in clinical variables among the patients were analyzed. We constructed a predictive model using pathological imaging features and validated its performance on the validation set. Its accuracy was evaluated using the area under the curve (AUC). Moreover, a calibration curve was used to assess its calibration and a decision curve was generated to estimate its net benefit.

### Survival analysis, GSEA, immune microenvironment analysis, and TMB analysis

We used the proposed model to generate prediction scores (PSes) for all H&E-stained images. To classify patients into high- and low-PS groups, we employed the survminer R package and Cutoff Finder web application to determine suitable cutoff values. Then, survival analysis, gene set enrichment analysis (GSEA), immune microenvironment analysis, and mutation analysis were conducted. For survival analysis, the Kaplan–Meier survival curve was plotted using the survival R package. The GSEA subroutine of the clusterProfiler *R* package was used for GSEA against the KEGG Gene Set Collection and Hallmark Gene Set Collection. Gene expression data were uploaded to the CIBERSORTx online platform. Immune infiltration in breast cancer samples was quantified using the CIBERSORTx algorithm. Immune cells include *T*, *B*, and *NK*, dendritic, and mast cells, as well as macrophages, eosinophils, and neutrophils. Mutation annotation format files provided on the TCGA database’s data portal for breast cancer were downloaded for tumor mutational burden (TMB) analysis. The calculation and visualization of the overall TMB were conducted in *R* using the maftools package.

### Statistical analysis

To evaluate the associations between IDO1/PS and various clinical and pathological factors (such as age, TNM stage, ER, PR, HER2, margin status, histologic type, and treatment type), we employed either the Χ-square test or Fisher's exact test. Wilcoxon rank-sum test was performed to determine the differences between the two groups. The log-rank test was used for Kaplan–Meier survival analysis. Additionally, the impact of the selected variables on OS was determined through both univariate and multivariate Cox regression analyses. To calculate correlations, spearman-rank correlation analysis was used. A *p*-value < 0.05 was considered to indicate statistical significance.

## Results

### Relationships between IDO1 expression, clinical variables, and survival

**Fig. 1:**
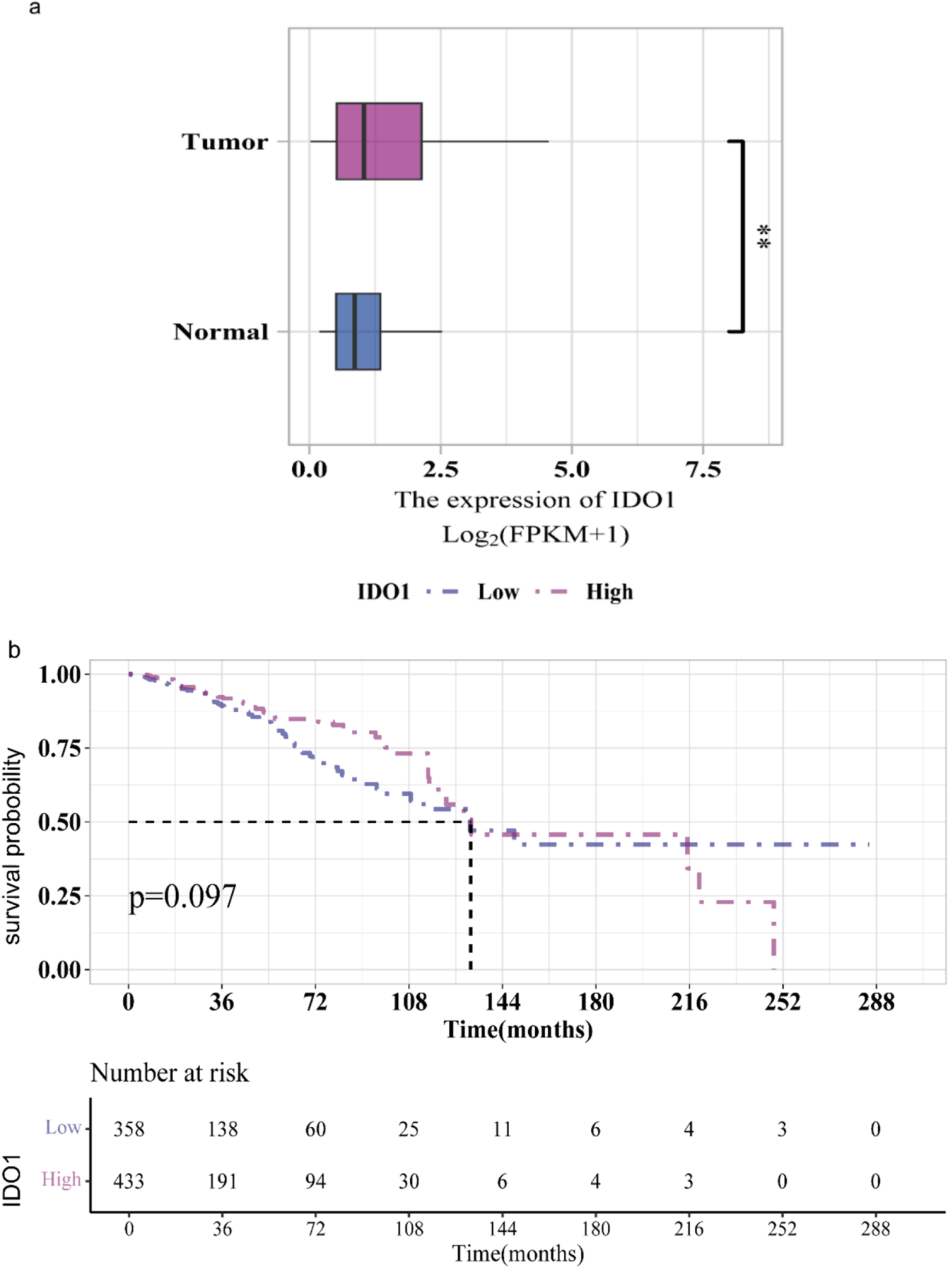
Survival analysis of IDO1: **a** Difference in IDO1 expression between the tumor group and normal group; **b** Kaplan–Meier survival plot revealing no significant difference in OS between the tumor group and normal group. **p < 0.01

**Fig. 2:**
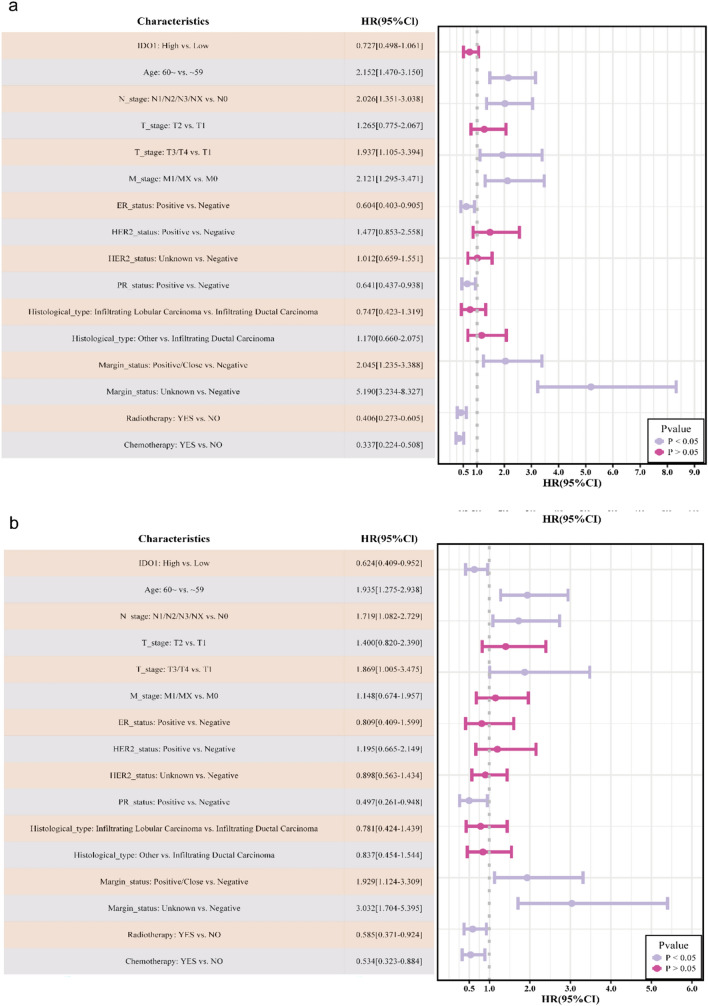
Univariate and multivariate logistic regression analysis: **a** Univariate logistic regression analysis did not reveal any significant difference in OS between the IDO1-low and IDO1-high groups; **b** Multivariate logistic regression analysis revealed that IDO1 was associated with OS

To assess the clinical significance of IDO1 in breast cancer, we investigated the associations between IDO1 expression, clinicopathological variables, and OS. Patients were divided into two groups based on their IDO1 levels: high (*n* = 433) and low (*n* = 358). The cut-off value of 0.9747 was determined using the survminer package. Notably, we found significant differences in *T*-stage, HER2_status, hormone receptor status, as well as histologic types and treatment types between the low- and high-IDO1 groups (all* p* < 0.05, see Supplementary Table 1). Additionally, the tumor group exhibited higher IDO1 expression levels than the normal group *(p* < 0.01, Fig. 1a). Moreover, neither Kaplan–Meier curves (Fig. 1b) nor univariate logistic regression analysis (Fig. 2a) revealed any significant differences in OS between the two groups. However, after adjustment, multivariate Cox regression analysis (Fig. 2b and Supplementary Table 2) indicated that high IDO1 expression was a favorable prognostic factor for OS (hazard ratio [HR] = 0.624, 95% confidence interval [CI] 0.409–0.952, *p* = 0.029). Subgroup analyses revealed no significant interactions between clinical variables in terms of OS (*p* > 0.05, see Supplementary Figure S2).

### Pathomic feature extraction and selection

**Fig. 3:**
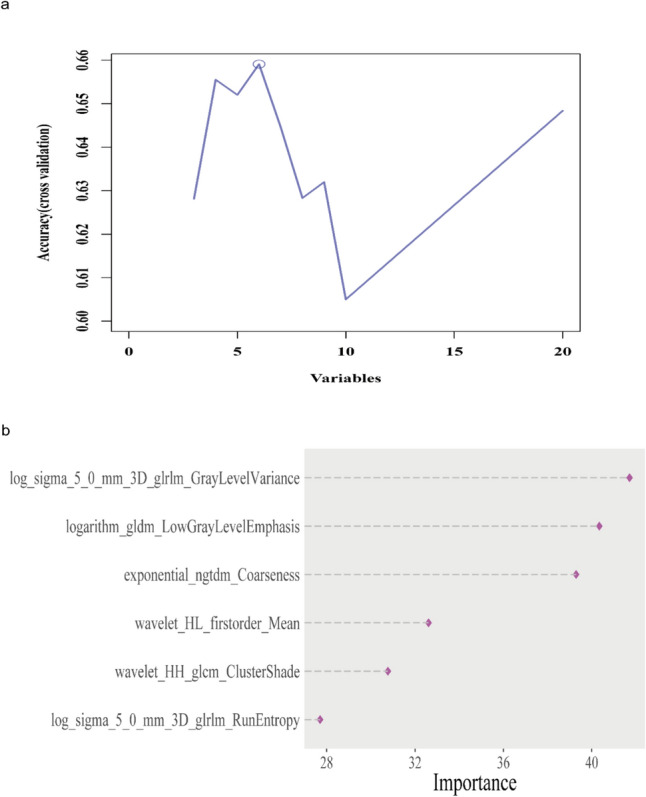
Optimal features selected by mRMR-RFE: **a** Accuracy of the first six features; **b** Importance of the six features in the GBM algorithm

Considering the clinical importance of IDO1, our aim was to develop a pathomic model capable of predicting IDO1 expression. Data of 791 patients were randomly classified into training (*n* = 555, 70%) and validation (*n* = 236, 30%) sets. The patients in both sets had similar statistics in terms of their clinical and pathological characteristics (*p* > 0.05; Supplementary Table 3); hence, the two sets were comparable. Following the image segmentation and feature extraction process, 10 sub-images were randomly selected, and 1,488 imaging features were extracted from each sub-image. Subsequently, we calculated the mean values of the 10 sub-images. Our study aimed to determine the optimal predictive feature combination to construct a model for breast cancer. First, the mRMR technique was applied to eliminate redundant and irrelevant features, and the top 20 features were retained. Second, we applied RFE to select the optimal features among the 20 mRMR features, and six features were identified (Fig. 3a). Figure 3b shows the importance of these six features.

### Construction and validation of a pathomic model

**Fig. 4:**
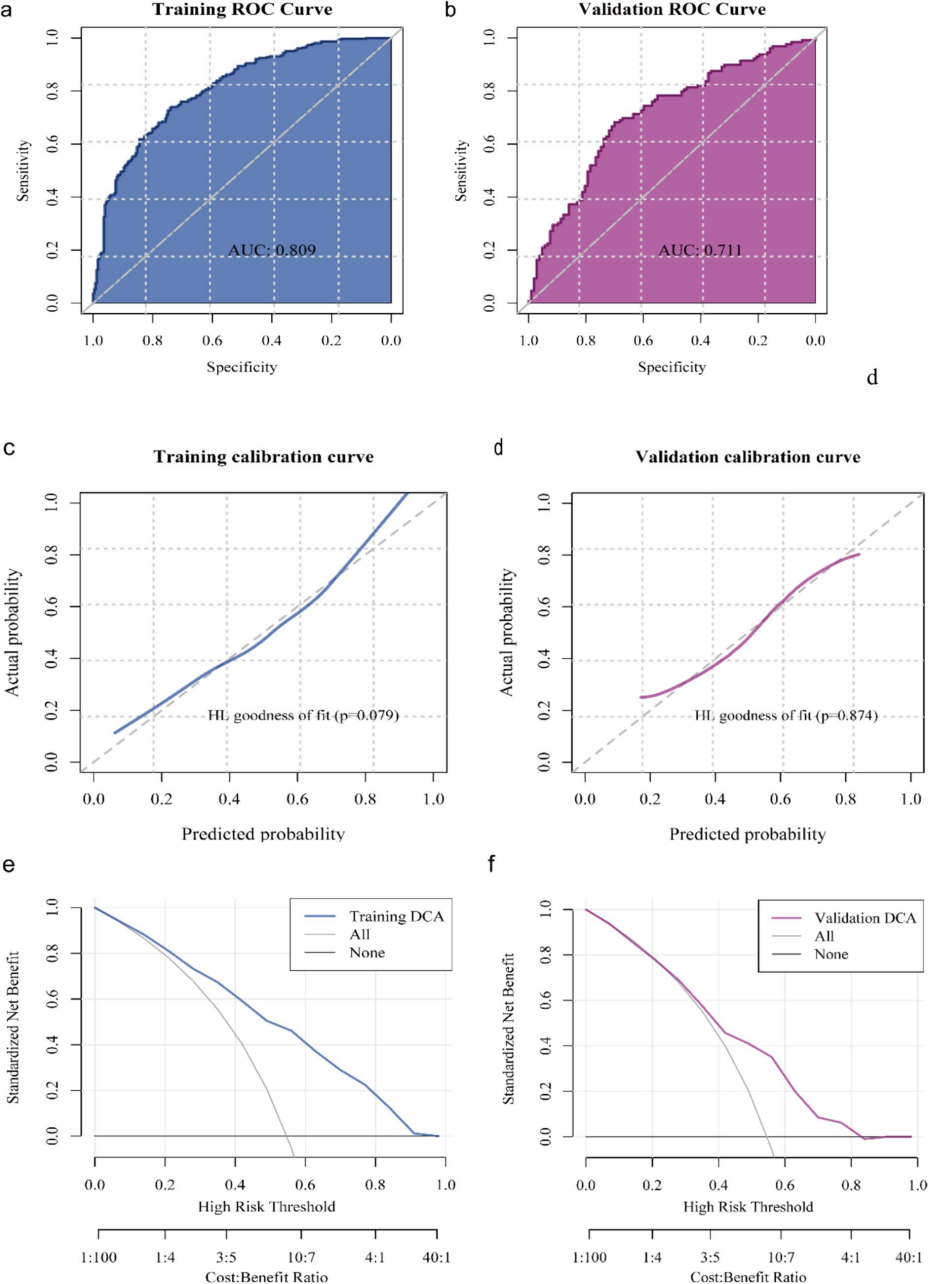
Evaluation of the predictive performance of the pathomic model: **a** AUC on the training set; **b** AUC on the validation set; **c** Calibration curve analysis of the training set; **d** Calibration curve analysis of the validation set; **e** DCA of the training set; and **f** DCA of the validation set. AUC: area under the curve, DCA: decision curve analysis

Using the six pathological features described in the previous section, we developed a predictive model through the GBM algorithm based on the training set. To assess the predictive performance of the model in breast cancer, receiver operating characteristic curve, calibration and decision curves were plotted for the training and validation sets. As Fig. 4a and 4b show, the model performed well in predicting IDO1 expression (AUC = 0.809 for the training set and AUC = 0.711 for the validation set). From the calibration curves, this model showed a high degree of fit for IDO1 expression prediction compared to the actual IDO1 expression levels (*p* > 0.05, see Fig. 4c and 4d). Furthermore, decision curve analysis (DCA) revealed that the model offers a significant net benefit for predictions (Fig. 4e and 4f). These results suggest that our model based on HE slices can predict IDO1 expression.

### PS and clinicopathological variables, OS

**Fig. 5:**
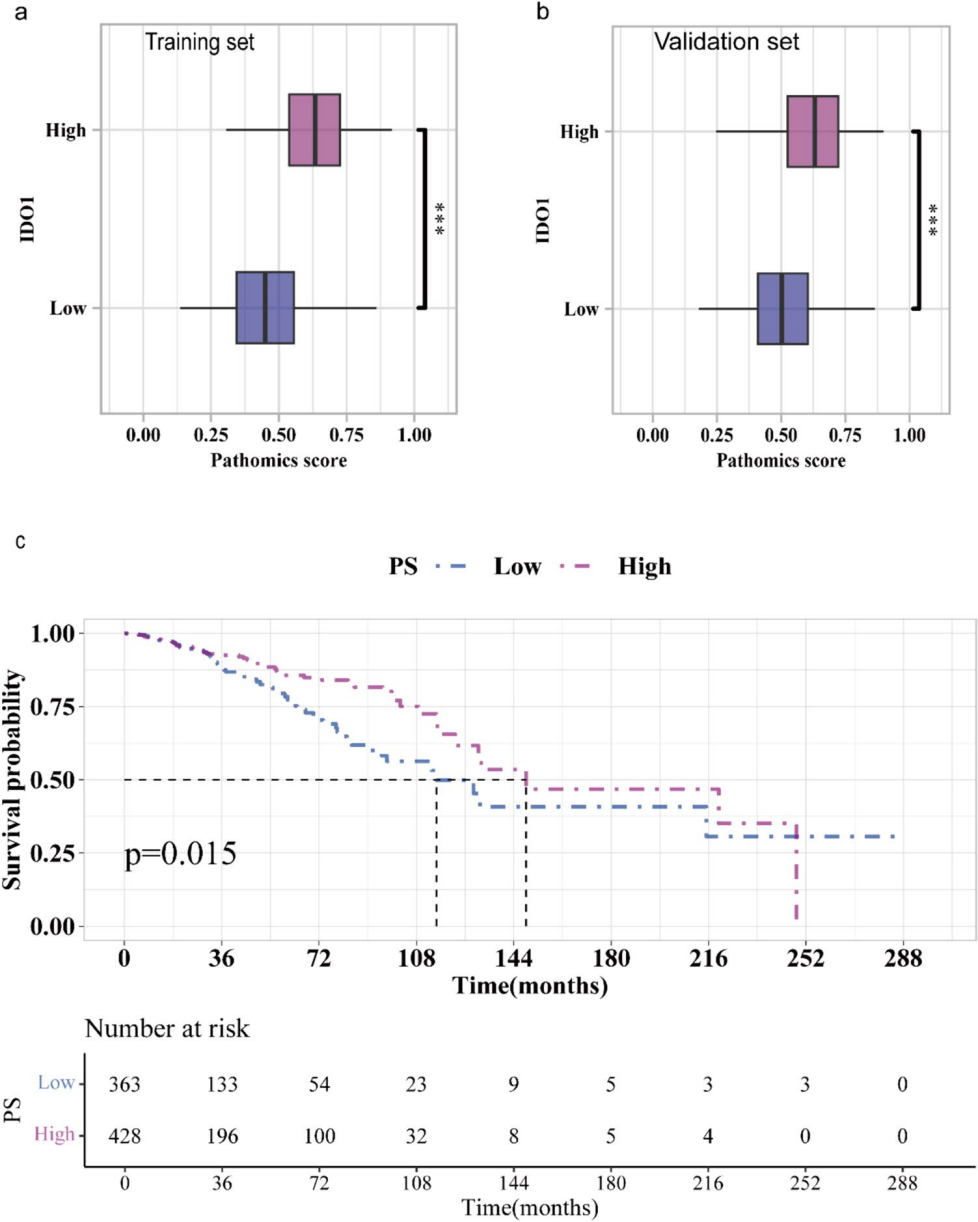
Survival analysis based on the PS: **a** Difference in PS between the training and validation sets; **b** Kaplan–Meier survival plot revealing a significant difference in OS between the PS-low and PS-high groups.***p < 0.001

**Fig. 6:**
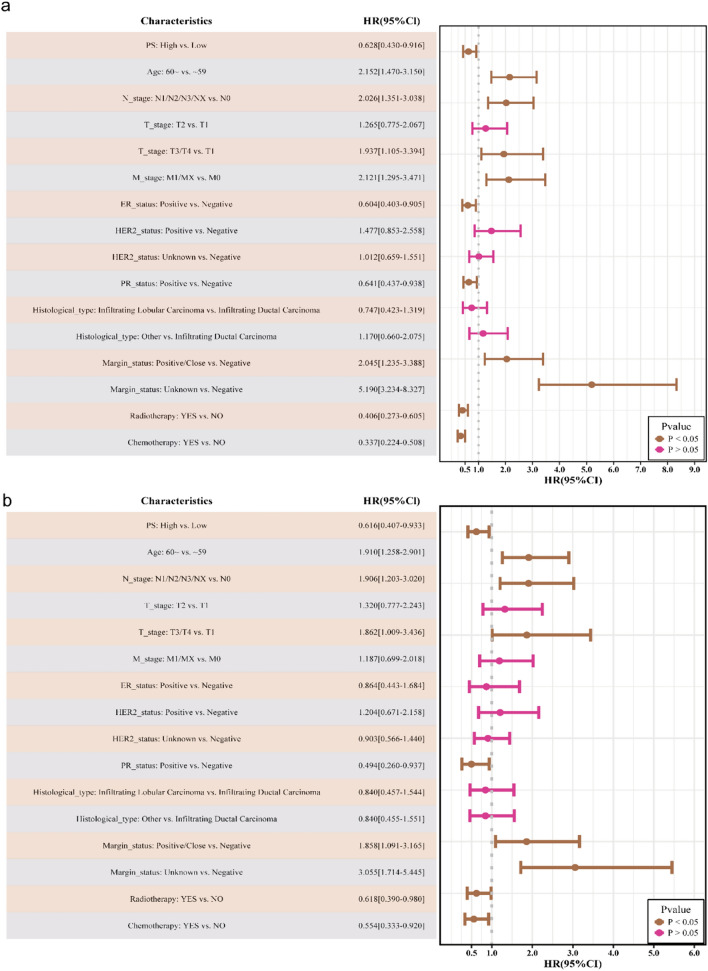
High PS is a favorable prognostic factor in patients with breast cancer **a** Univariate logistic regression analysis revealing a significant difference in OS between the PS-low and PS-high groups; **b** Multivariate logistic regression analysis revealing that the PS was associated with OS

We further assessed the ability of the model to predict the prognosis of breast cancer in patients. Based on the cutoff value (0.5453) for PS, we separated the patient into high- or low-PS groups. As shown in Fig. 5a and 5b, patients in the high-IDO1 group exhibited a higher PS than those in the low-IDO1 group (*p* < 0.001). The low- and high-PS groups showed differences in age, hormone receptor status, as well as histological and treatment types (all* p* < 0.05; Table 1). Kaplan–Meier analysis (Fig. 5c) revealed that an elevated PS was associated with favorable OS (*p* = 0.015), and univariate and multivariate Cox regression analyses (Fig. 6a and 6b, see Supplementary Table 4) revealed that PS was an independent favorable factor for OS (HR = 0.616; 95% CI 0.407–0.933; *p* = 0.022). Subgroup analyses revealed no significant interaction between PS and OS among the clinical variables (*p* > 0.05; Supplementary Fig. S3). These results suggest that our model, which was trained to predict IDO1 expression, can also predict patients' OS.

**Table 1:** Correlation between PS values and clinicopathological variables in 791 patients with breast cancer extracted from the TCGA database

Variables	Total (*n* = 791)	Low (*n* = 363)	High (*n* = 428)	*p*
*Age, n (%)*				**0.045**
~ 59	439 (55)	187 (52)	252 (59)	
60 ~	352 (45)	176 (48)	176 (41)	
*N_stage, n (%)*				0.086
N0	365 (46)	180 (50)	185 (43)	
N1/N2/N3/NX	426 (54)	183 (50)	243 (57)	
*T_stage, n (%)*				0.61
T1	192 (24)	92 (25)	100 (23)	
T2	466 (59)	207 (57)	259 (61)	
T3/T4	133 (17)	64 (18)	69 (16)	
*M_stage, n (%)*				0.517
M0	665 (84)	309 (85)	356 (83)	
M1/MX	126 (16)	54 (15)	72 (17)	
*ER_status, n (%)*				** < 0.001**
Negative	182 (23)	59 (16)	123 (29)	
Positive	609 (77)	304 (84)	305 (71)	
*HER2_status, n (%)*				0.072
Negative	415 (52)	182 (50)	233 (54)	
Positive	133 (17)	55 (15)	78 (18)	
Unknown	243 (31)	126 (35)	117 (27)	
*PR_status, n (%)*				** < 0.001**
Negative	259 (33)	90 (25)	169 (39)	
Positive	532 (67)	273 (75)	259 (61)	
*Histological_type, n (%)*				**0.048**
Infiltrating Ductal Carcinoma	574 (73)	255 (70)	319 (75)	
Infiltrating Lobular Carcinoma	143 (18)	64 (18)	79 (18)	
Other	74 (9)	44 (12)	30 (7)	
*Margin_status, n (%)*				0.681
Negative	665 (84)	301 (83)	364 (85)	
Positive/Close	79 (10)	38 (10)	41 (10)	
Unknown	47 (6)	24 (7)	23 (5)	
*Radiotherapy, n (%)*				0.128
NO	381 (48)	186 (51)	195 (46)	
YES	410 (52)	177 (49)	233 (54)	
*Chemotherapy, n (%)*				**0.001**
NO	332 (42)	175 (48)	157 (37)	
YES	459 (58)	188 (52)	271 (63)	

The bold values mean the clinical variable with statistically significance

### PS and pathways, immune microenvironment, TMB

**Fig. 7:**
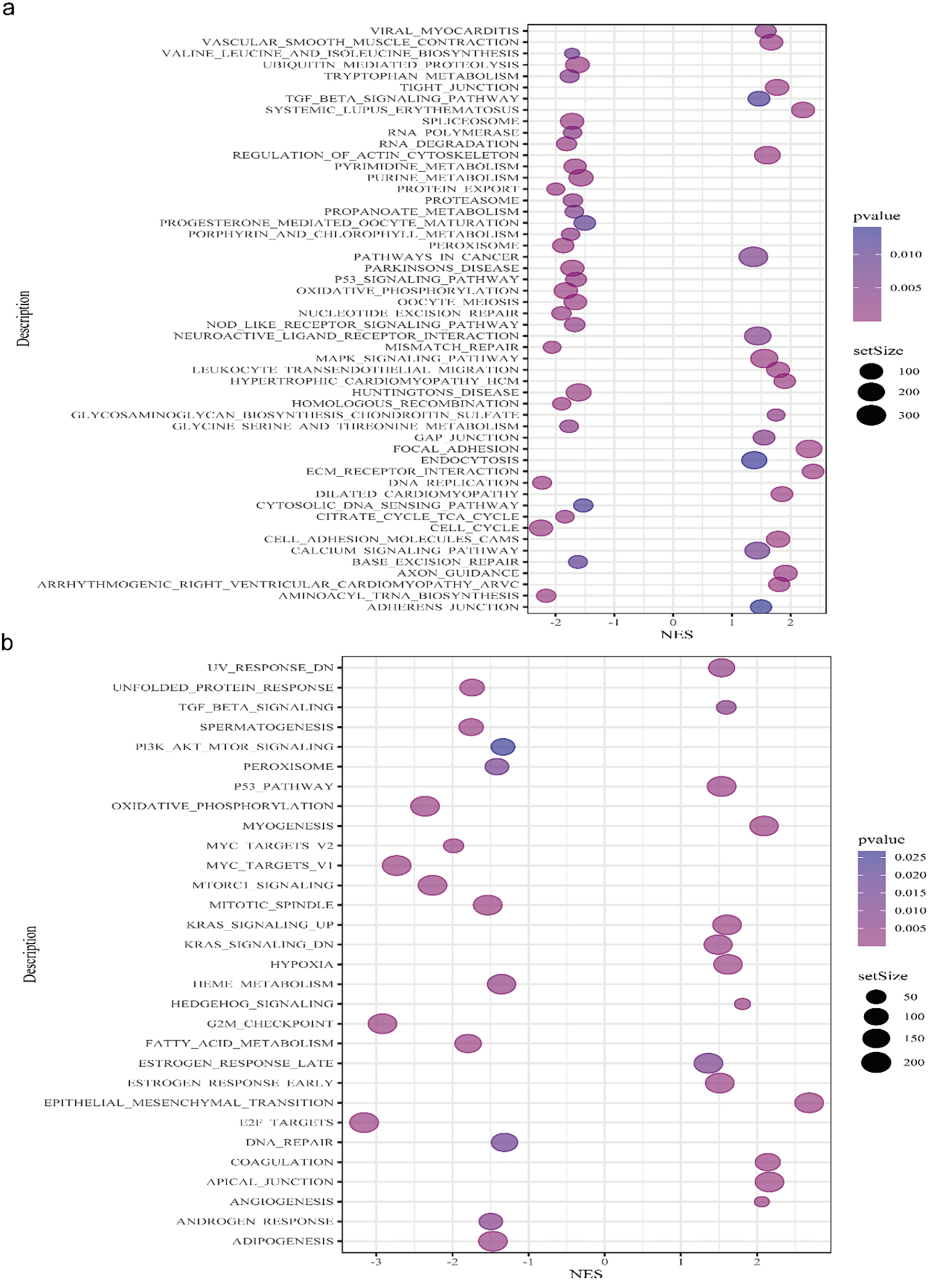
GSEA results: **a** Enriched pathways in the KEGG pathway gene sets (*p* < 0.05, *q* < 0.25); **b** Enriched pathways in the Hallmarks of Cancer gene sets (*p* < 0.05, *q* < 0.25)

To explore the potential molecular mechanism, we performed GSEA on differentially expressed genes in patients with high and low PS. GSEA identified changes in 52 pathways annotated in the KEGG pathway gene sets (Fig. 7a) and 30 pathways in the Hallmarks of Cancer gene sets (Fig. 7b). We found that these differentially expressed genes were involved in KEGG oxidative phosphorylation, transforming growth factor β (TGF-β) signaling, extracellular matrix (ECM)-receptor interaction, peroxisome, p53 signal pathway, tryptophan metabolism, and cell junction (i.e., cell adhesion molecules, adherent junction, focal adhesion) and overlapped with hallmark oxidative phosphorylation, peroxisome, p53 signal pathway, epithelial-mesenchymal transition (EMT), heme metabolism, and adipogenesis.

**Fig. 8:**
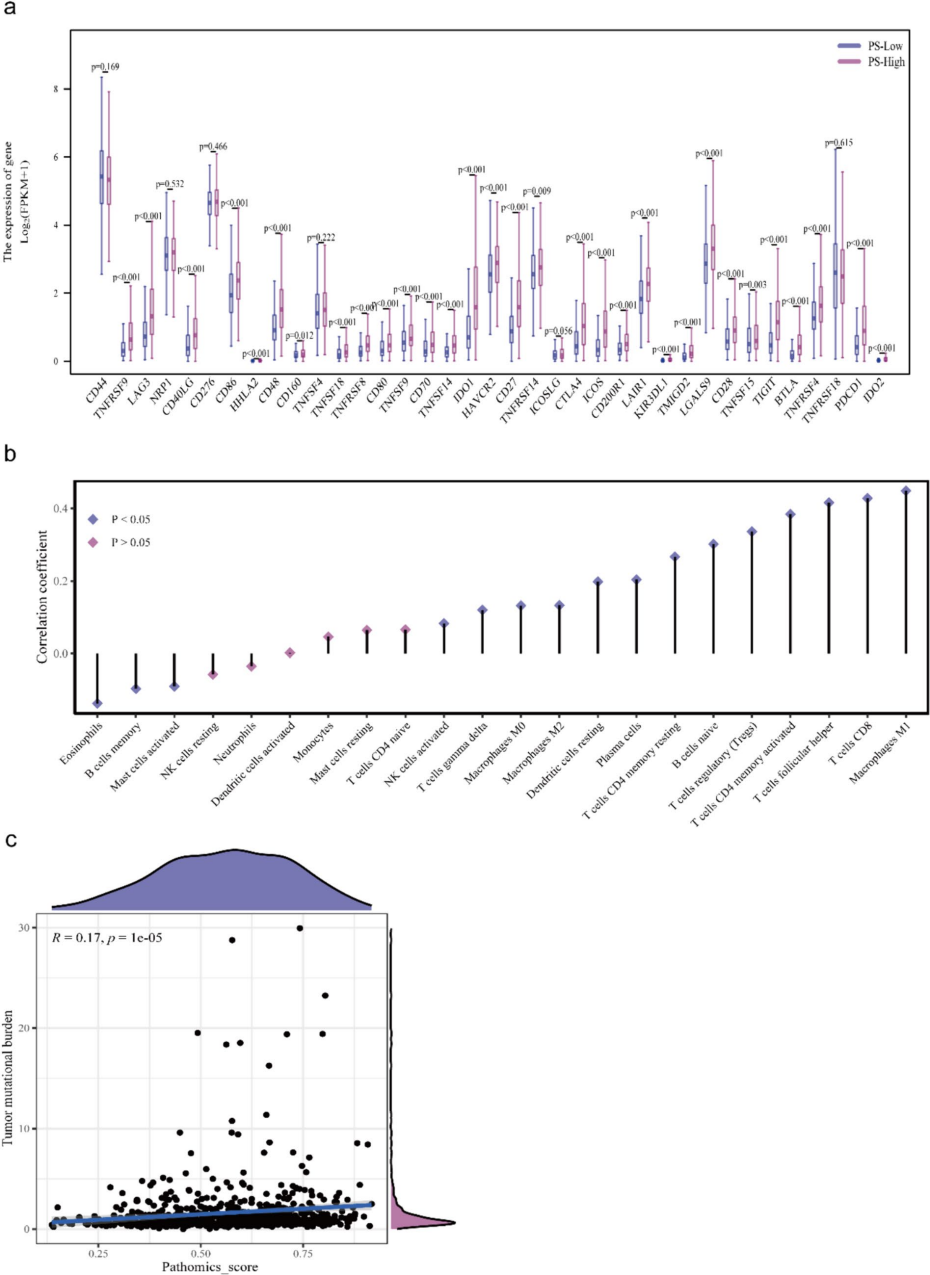
Potential molecular mechanisms: **a** PS and the immune checkpoint genes; **b** PS and the tumor-infiltrating immune cells; and c) PS and TMB status

We further evaluated whether our model could guide patient immunotherapy. Figure 8a presents the variations in the expressions of genes associated with the immune system. The high-PS group exhibits considerably higher TIGIT, BTLA, ICOS, and PDCD1 expressions (*p* < 0.001). Figure 8b presents the relationship between the landscape of PS and the immune cell infiltration of the tumor. Notably, strong correlations between PS and M1 macrophages, CD8^+^ T cells, and *T* cell follicular helper cells were discovered. Moreover, Fig. 8c indicates a positive correlation between PS and TMB (*r* = 0.17, *p* < 0.001).

## Discussion

In this study, a pathomic model based on ML was constructed to predict IDO1 status and its relationship with prognosis directly from the six pathomic features of H&E-stained breast cancer sections. It was determined that IDO1 expression and PS were upregulated and associated with a better prognosis. The pathomic model yielded an accurate overall prediction performance. A higher PS was correlated with higher immune checkpoints expression, tumor-infiltrating immune cell levels and TMB.

It was observed that IDO1 expression and PS levels were elevated in breast cancer patients. These observations were consistent with those of previous studies. Higher IDO1 expression has been observed in breast cancer [6, 11, 27] and other cancers [11], including colorectal cancer, esophageal carcinoma, cervical squamous cell carcinoma, melanoma, and pancreatic cancer. Multiple reports [23–26] have described ongoing and promising efforts aimed at predicting biomarker status in breast cancer using ML-based H&E image analysis. These studies focused on tissue microarray datasets, whereas this study focused on RNA-seq data from TCGA to avoid the disadvantages of the IHC method. The enrichment analysis indicated that pathways such as ECM-receptor interaction, TGF-β signaling, angiogenesis, tryptophan metabolism, heme metabolic processes, EMT, and cell junction should be considered. TGF-β signaling, which is regulated by the ECM, induces EMT, resulting in the disintegration of cellular junctions, reconfiguration of the cytoskeletal architecture, and modifications of cellular polarity and morphology, all of which can potentially cause distinctive histopathological changes in images [28].

Elevated IDO1 expression and PS were positively correlated with a better prognosis in breast cancer. Prior studies have consistently shown that high IDO1 expression levels are correlated with favorable outcomes in breast [6, 27] and other cancers [29, 30]. However, certain studies found that a high IDO1 level is associated with a poor prognosis [31]. Therefore, the intricate relationship between IDO1 levels and clinical outcomes can be attributed to various factors such as age, sex, tumor type [6], follow-up time, study quality, IDO1/CD8 ratio [5], and IDO1 expression site [29]. Considering the strong heterogeneity of tumors, pathomic features based on H&E images could provide more comprehensive and objective details corresponding to pathological factors, such as tumor proliferation, angiogenesis, tumor necrosis, and tumor immunity. Histopathological image features have been extensively employed in numerous past investigations for survival prediction in various cancers, including hepatocellular carcinoma [32], head and neck squamous cell carcinoma [33], lung adenocarcinoma [34], colorectal cancer [35], colon adenocarcinoma [36], and glioma [37]. By adopting a similar digital workflow, a pathomic model was constructed that outputs PS values. PS is correlated with favorable OS and is an independent protective factor. Furthermore, immune microenvironment and TMB analyses indicated that a high PS was positively associated with 32 immune checkpoints, including PD-1, CTLA-4, and LAG-3, as well as relatively high immune cell infiltration and TMB. The tumor microenvironment plays a crucial role in the development of both primary and acquired resistance to breast cancer immunotherapy [38]. It was speculated that although patients with high PS had a higher immune cell invasion and tumor mutation load, they might also have a weak immune response due to the higher expression of immune checkpoint-related genes. Patients with a high PS might derive more benefits from treatment with immune checkpoint inhibitors. Therefore, PS potentially plays a crucial role in facilitating the stratification of breast cancer patients for managing treatment. This suggests that healthcare professionals may use PS as a biomarker to improve prognosis predictions of breast cancer patients and select patients who would benefit more from IDO1 inhibitor immunotherapy.

The encouraging progress of ML methods and implementation of digital workflows in histopathology is noteworthy. These technological innovations have allowed the analysis of cancer biomarkers to be conducted on the slide-image level in many cases. The proposed pathomic model based on histological image features obtained through digital workflows could provide a new means of studying biomarker status conveniently, cheaply, robustly, and objectively, with high efficiency, accuracy, and generalizability.

This study had some limitations. Although the proposed pathomic model demonstrated significant predictive value, external validation studies and multicenter studies are necessary to verify its accuracy and practicability. In addition, the specific molecular mechanisms in this model are not well understood, and further investigation is required. Finally, as with any data-driven approach, the conclusions of the analysis are dependent on the accuracy of the initial input data.

## Conclusion

This study demonstrated that a pathomic model based on ML and histopathological image features could predict IDO1 status and prognosis in breast cancer patients. High IDO1/PS was found to correlate with favorable OS, and patients with high IDO1/PS might benefit more from treatment with immune checkpoint inhibitors. The findings might offer valuable insights for healthcare providers to determine appropriate treatment strategies for patients with breast cancer, demonstrating that machine learning approaches, together with histological images and RNA-seq data, would be of significant value.

### Supplementary Information

Below is the link to the electronic supplementary material.

Supplementary file1 (DOCX 705 kb)

## Data Availability

The datasets used in this study are available from the corresponding author upon reasonable request.
