# Supplementary material for: Pathomic model based on histopathological features and machine learning to predict IDO1 status and its association with breast cancer prognosis
Source: Breast Cancer Res Treat. 2024 May 23;207(1):151–65. doi: 10.1007/s10549-024-07350-6 (PMC11230954; doi:10.1007/s10549-024-07350-6)
Supplement: Supplementary file 1 — Supplementary file1 (DOCX 705 kb) [file 10549_2024_7350_MOESM1_ESM.docx]

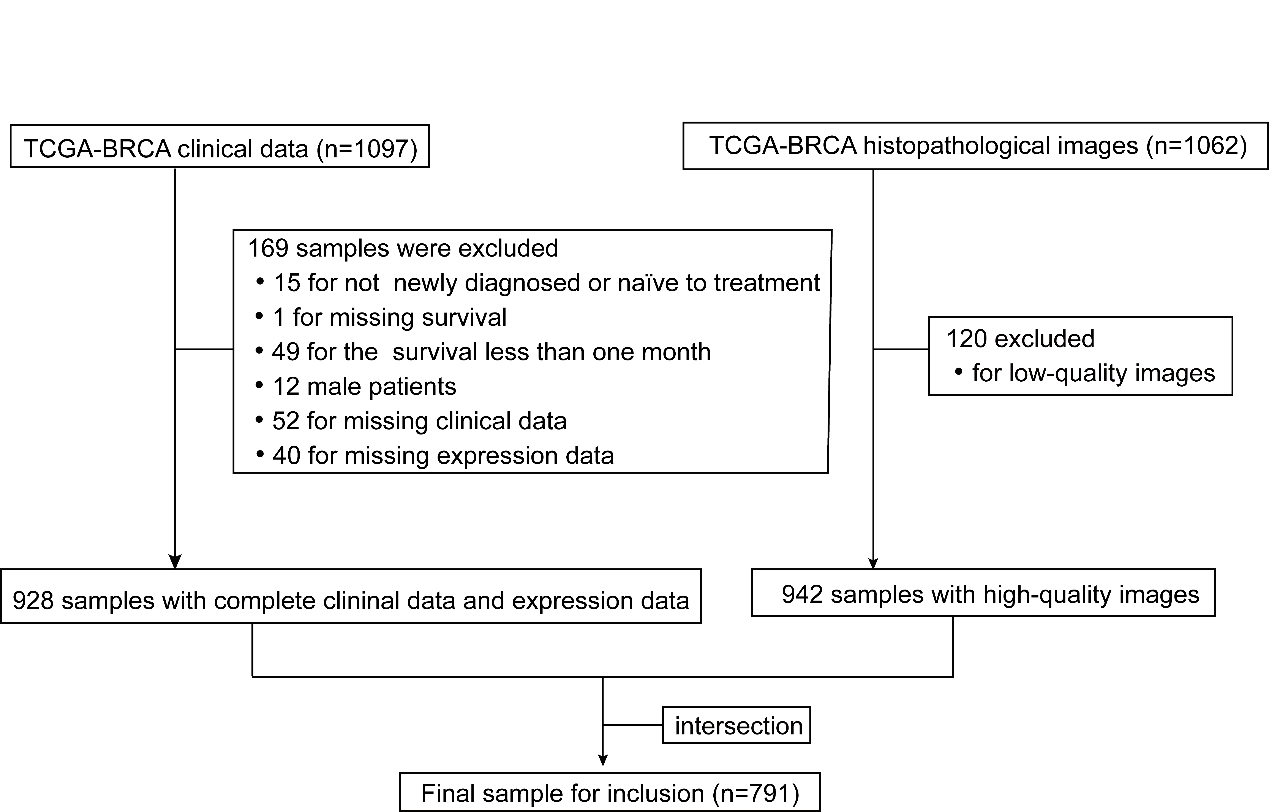


Supplementary Fig.S1 Flow diagram showing the inclusion and exclusion criteria.


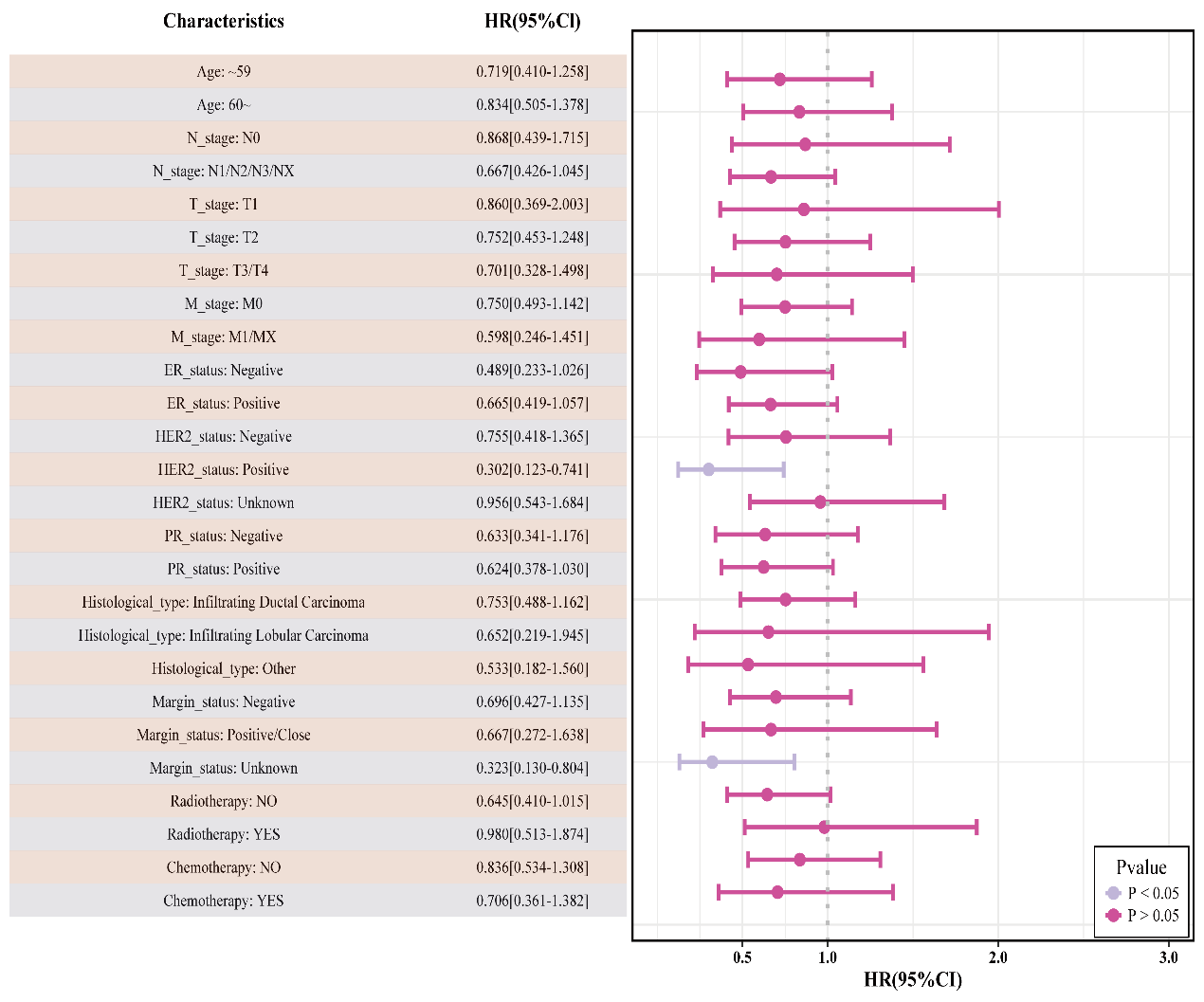


Supplementary Fig. S2 subgroup analysis based on IDO1


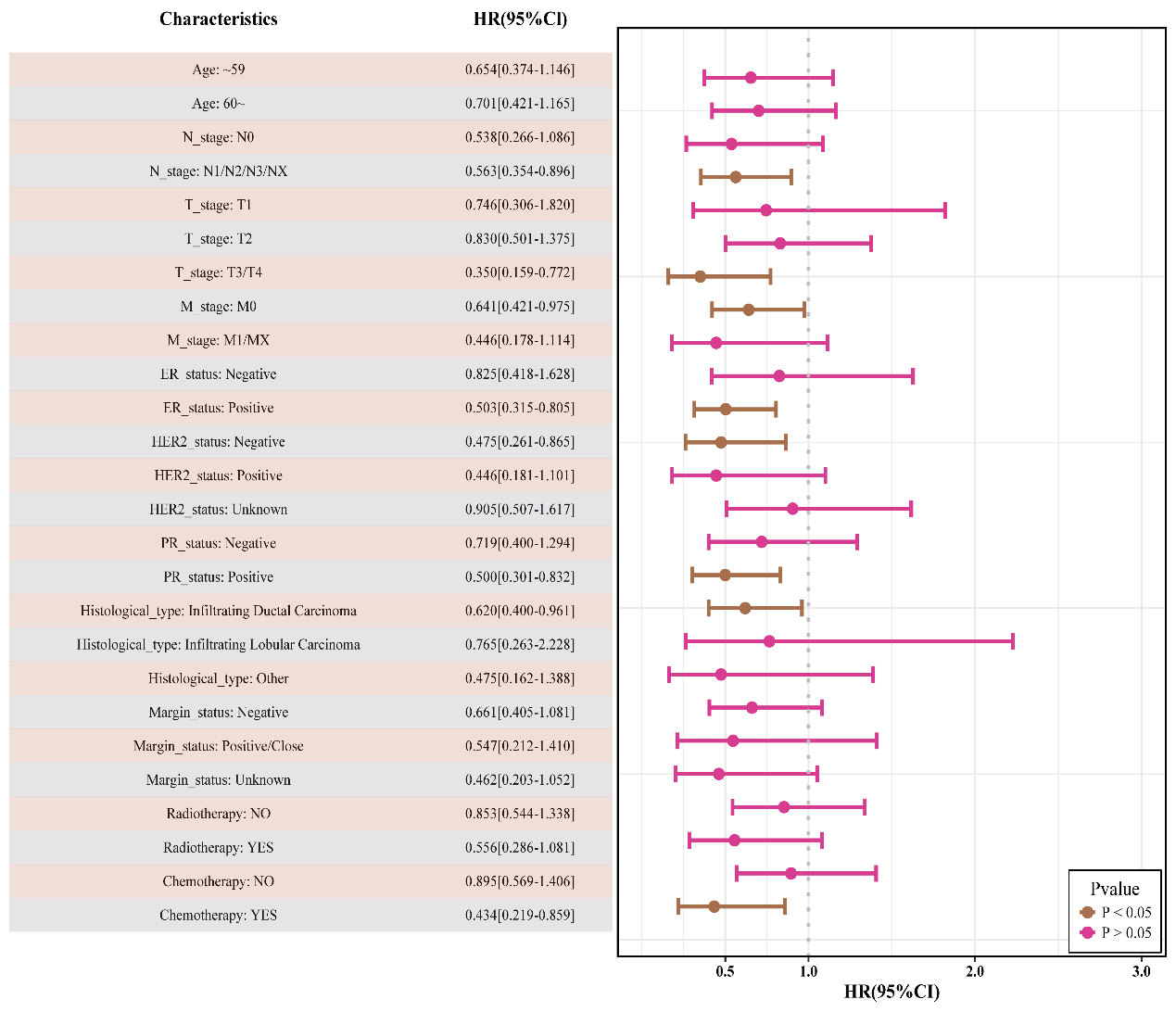


Supplementary Fig. S3 subgroup analysis based on PS

Supplementary Table 1 Correlation between IDO1 expression and clinicopathological variables in 791 BRCA patients extracted from TCGA database

| **Variables** | **Total (n = 791)** | **Low (n = 358)** | **High (n = 433)** | **p** |
| --- | --- | --- | --- | --- |
| Age, n (%) |  |  |  | 0.08 |
| ~59 | 439 (55) | 186 (52) | 253 (58) |  |
| 60~ | 352 (45) | 172 (48) | 180 (42) |  |
| N_stage, n (%) |  |  |  | 0.921 |
| N0 | 365 (46) | 164 (46) | 201 (46) |  |
| N1/N2/N3/NX | 426 (54) | 194 (54) | 232 (54) |  |
| T_stage, n (%) |  |  |  | **0.015** |
| T1 | 192 (24) | 94 (26) | 98 (23) |  |
| T2 | 466 (59) | 192 (54) | 274 (63) |  |
| T3/T4 | 133 (17) | 72 (20) | 61 (14) |  |
| M_stage, n (%) |  |  |  | 1 |
| M0 | 665 (84) | 301 (84) | 364 (84) |  |
| M1/MX | 126 (16) | 57 (16) | 69 (16) |  |
| ER_status, n (%) |  |  |  | **<** **0.001** |
| Negative | 182 (23) | 38 (11) | 144 (33) |  |
| Positive | 609 (77) | 320 (89) | 289 (67) |  |
| HER2_status, n (%) |  |  |  | **0.014** |
| Negative | 415 (52) | 187 (52) | 228 (53) |  |
| Positive | 133 (17) | 47 (13) | 86 (20) |  |
| Unknown | 243 (31) | 124 (35) | 119 (27) |  |
| PR_status, n (%) |  |  |  | **< 0.001** |
| Negative | 259 (33) | 72 (20) | 187 (43) |  |
| Positive | 532 (67) | 286 (80) | 246 (57) |  |
| Histological_type, n (%) |  |  |  | **0.026** |
| Infiltrating Ductal Carcinoma | 574 (73) | 247 (69) | 327 (76) |  |
| Infiltrating Lobular Carcinoma | 143 (18) | 67 (19) | 76 (18) |  |
| Other | 74 (9) | 44 (12) | 30 (7) |  |
| Margin_status, n (%) |  |  |  | 0.434 |
| Negative | 665 (84) | 295 (82) | 370 (85) |  |
| Positive/Close | 79 (10) | 38 (11) | 41 (9) |  |
| Unknown | 47 (6) | 25 (7) | 22 (5) |  |
| Radiotherapy, n (%) |  |  |  | **0.022** |
| NO | 381 (48) | 189 (53) | 192 (44) |  |
| YES | 410 (52) | 169 (47) | 241 (56) |  |
| Chemotherapy, n (%) |  |  |  | **< 0.001** |
| NO | 332 (42) | 178 (50) | 154 (36) |  |
| YES | 459 (58) | 180 (50) | 279 (64) |  |

*The bold values mean the clinical variable with statistically significance.*

Supplementary Table 2 Univariate and multivariate analysis for OS of 791 BRCA patients from the TCGA database

|  | **Univariate analysis** |  | **Multivariate analysis** | |
| --- | --- | --- | --- | --- |
| **variables** | **HR (95% CI)** | **P value** | **HR (95% CI)** | **P value** |
| IDO1: High vs. Low | 0.727(0.498-1.061) | 0.098 | 0.624(0.409-0.952) | **0.029** |
| Age: 60~ vs. ~59 | 2.152(1.47-3.15) | < 0.001 | 1.935(1.275-2.938) | **0.002** |
| N_stage: N1/N2/N3/NX vs. N0 | 2.026(1.351-3.038) | < 0.001 | 1.719(1.082-2.729) | **0.022** |
| T_stage: T2 vs. T1 | 1.265(0.775-2.067) | 0.347 | 1.4(0.82-2.39) | 0.218 |
| T_stage: T3/T4 vs. T1 | 1.937(1.105-3.394) | 0.021 | 1.869(1.005-3.475) | **0.048** |
| M_stage: M1/MX vs. M0 | 2.121(1.295-3.471) | 0.003 | 1.148(0.674-1.957) | 0.611 |
| ER_status: Positive vs. Negative | 0.604(0.403-0.905) | 0.015 | 0.809(0.409-1.599) | 0.541 |
| HER2_status: Positive vs. Negative | 1.477(0.853-2.558) | 0.164 | 1.195(0.665-2.149) | 0.551 |
| HER2_status: Unknown vs. Negative | 1.012(0.659-1.551) | 0.958 | 0.898(0.563-1.434) | 0.653 |
| PR_status: Positive vs. Negative | 0.641(0.437-0.938) | 0.022 | 0.497(0.261-0.948) | **0.034** |
| Histological_type: Infiltrating Lobular Carcinoma vs. Infiltrating Ductal Carcinoma | 0.747(0.423-1.319) | 0.314 | 0.781(0.424-1.439) | 0.427 |
| Histological_type: Other vs. Infiltrating Ductal Carcinoma | 1.17(0.66-2.075) | 0.591 | 0.837(0.454-1.544) | 0.57 |
| Margin_status: Positive/Close vs. Negative | 2.045(1.235-3.388) | 0.005 | 1.929(1.124-3.309) | **0.017** |
| Margin_status: Unknown vs. Negative | 5.19(3.234-8.327) | < 0.001 | 3.032(1.704-5.395) | **< 0.001** |
| Radiotherapy: YES vs. NO | 0.406(0.273-0.605) | < 0.001 | 0.585(0.371-0.924) | **0.021** |
| Chemotherapy: YES vs. NO | 0.337(0.224-0.508) | < 0.001 | 0.534(0.323-0.884) | **0.015** |

*HR, hazard ratio; 95% CI: 95% confidence interval; OS, overall survival.*

*The bold values mean the clinical variable with statistically significance.*

Supplementary Table 3 The difference of clinical variables between the two data sets

| Variables | Total (n = 791) | Train (n = 555) | Validation (n = 236) | p |
| --- | --- | --- | --- | --- |
| IDO1, n (%) |  |  |  | 1 |
| Low | 358 (45) | 251 (45) | 107 (45) |  |
| High | 433 (55) | 304 (55) | 129 (55) |  |
| Age, n (%) |  |  |  | 1 |
| ~59 | 439 (55) | 308 (55) | 131 (56) |  |
| 60~ | 352 (45) | 247 (45) | 105 (44) |  |
| N_stage, n (%) |  |  |  | 0.19 |
| N0 | 365 (46) | 265 (48) | 100 (42) |  |
| N1/N2/N3/NX | 426 (54) | 290 (52) | 136 (58) |  |
| T_stage, n (%) |  |  |  | 0.885 |
| T1 | 192 (24) | 136 (25) | 56 (24) |  |
| T2 | 466 (59) | 328 (59) | 138 (58) |  |
| T3/T4 | 133 (17) | 91 (16) | 42 (18) |  |
| M_stage, n (%) |  |  |  | 0.685 |
| M0 | 665 (84) | 469 (85) | 196 (83) |  |
| M1/MX | 126 (16) | 86 (15) | 40 (17) |  |
| ER_status, n (%) |  |  |  | 0.184 |
| Negative | 182 (23) | 120 (22) | 62 (26) |  |
| Positive | 609 (77) | 435 (78) | 174 (74) |  |
| HER2_status, n (%) |  |  |  | 0.636 |
| Negative | 415 (52) | 288 (52) | 127 (54) |  |
| Positive | 133 (17) | 91 (16) | 42 (18) |  |
| Unknown | 243 (31) | 176 (32) | 67 (28) |  |
| PR_status, n (%) |  |  |  | 0.387 |
| Negative | 259 (33) | 176 (32) | 83 (35) |  |
| Positive | 532 (67) | 379 (68) | 153 (65) |  |
| Histological_type, n (%) |  |  |  | 0.078 |
| Infiltrating Ductal Carcinoma | 574 (73) | 393 (71) | 181 (77) |  |
| Infiltrating Lobular Carcinoma | 143 (18) | 102 (18) | 41 (17) |  |
| Other | 74 (9) | 60 (11) | 14 (6) |  |
| Margin_status, n (%) |  |  |  | 0.522 |
| Negative | 665 (84) | 467 (84) | 198 (84) |  |
| Positive/Close | 79 (10) | 58 (10) | 21 (9) |  |
| Unknown | 47 (6) | 30 (5) | 17 (7) |  |
| Radiotherapy, n (%) |  |  |  | 0.622 |
| NO | 381 (48) | 271 (49) | 110 (47) |  |
| YES | 410 (52) | 284 (51) | 126 (53) |  |
| Chemotherapy, n (%) |  |  |  | 0.576 |
| NO | 332 (42) | 237 (43) | 95 (40) |  |
| YES | 459 (58) | 318 (57) | 141 (60) |  |
| OS, n (%) |  |  |  | 0.262 |
| Alive | 682 (86) | 484 (87) | 198 (84) |  |
| Dead | 109 (14) | 71 (13) | 38 (16) |  |
| OS.time, Median (Q1,Q3) | 29.37 (16.17, 57.83) | 29.27 (16.7, 61.3) | 29.82 (14.7, 54.68) | 0.516 |

**Supplementary Table 4.** Univariate and multivariate analysis for OS of 791 BRCA patients from the TCGA database

|  | Univariate analysis |  | Multivariate analysis | |
| --- | --- | --- | --- | --- |
|  | HR (95% CI) | P value | HR (95% CI) | P value |
| PS: High vs. Low | 0.628(0.43-0.916) | **0.016** | 0.616(0.407-0.933) | **0.022** |
| Age: 60~ vs. ~59 | 2.152(1.47-3.15) | **< 0.001** | 1.91(1.258-2.901) | **0.002** |
| N_stage: N1/N2/N3/NX vs. N0 | 2.026(1.351-3.038) | **< 0.001** | 1.906(1.203-3.02) | **0.006** |
| T_stage: T2 vs. T1 | 1.265(0.775-2.067) | 0.347 | 1.32(0.777-2.243) | 0.304 |
| T_stage: T3/T4 vs. T1 | 1.937(1.105-3.394) | **0.021** | 1.862(1.009-3.436) | **0.047** |
| M_stage: M1/MX vs. M0 | 2.121(1.295-3.471) | **0.003** | 1.187(0.699-2.018) | 0.526 |
| ER_status: Positive vs. Negative | 0.604(0.403-0.905) | **0.015** | 0.864(0.443-1.684) | 0.668 |
| HER2_status: Positive vs. Negative | 1.477(0.853-2.558) | 0.164 | 1.204(0.671-2.158) | 0.534 |
| HER2_status: Unknown vs. Negative | 1.012(0.659-1.551) | 0.958 | 0.903(0.566-1.44) | 0.668 |
| PR_status: Positive vs. Negative | 0.641(0.437-0.938) | **0.022** | 0.494(0.26-0.937) | **0.031** |
| Histological_type: Infiltrating Lobular Carcinoma vs. Infiltrating Ductal Carcinoma | 0.747(0.423-1.319) | 0.314 | 0.84(0.457-1.544) | 0.575 |
| Histological_type: Other vs. Infiltrating Ductal Carcinoma | 1.17(0.66-2.075) | 0.591 | 0.84(0.455-1.551) | 0.577 |
| Margin_status: Positive/Close vs. Negative | 2.045(1.235-3.388) | **0.005** | 1.858(1.091-3.165) | **0.023** |
| Margin_status: Unknown vs. Negative | 5.19(3.234-8.327) | **< 0.001** | 3.055(1.714-5.445) | **< 0.001** |
| Radiotherapy: YES vs. NO | 0.406(0.273-0.605) | **< 0.001** | 0.618(0.39-0.98) | **0.041** |
| Chemotherapy: YES vs. NO | 0.337(0.224-0.508) | **< 0.001** | 0.554(0.333-0.92) | **0.022** |

*HR, hazard ratio; 95% CI: 95% confidence interval; OS, overall survival.*

*The bold values mean the clinical variable with statistically significance.*
